# Rapid and selective detection of macrocyclic trichothecene producing *Stachybotrys chartarum* strains by loop-mediated isothermal amplification (LAMP)

**DOI:** 10.1007/s00216-021-03436-y

**Published:** 2021-06-15

**Authors:** Johannes Köck, Christoph Gottschalk, Sebastian Ulrich, Karin Schwaiger, Manfred Gareis, Ludwig Niessen

**Affiliations:** 1 Faculty of Veterinary Medicine, Ludwig-Maximilians-University Munich, Schoenleutnerstr. 8, 85764, Oberschleissheim, Germany; 2 Institute for Infectious Diseases and Zoonoses, Faculty of Veterinary Medicine, Ludwig-Maximilians-University Munich, Veterinaerstraße 13, 80539 Munich, Germany; 3 Institute for Food Safety, Food Technology and Veterinary Public Health, Unit of Food Hygiene and Technology, University of Veterinary Medicine, Veterinärplatz 1, A-1210 Vienna, Austria; 4 TUM School of Life Sciences, Technical University of Munich, Gregor-Mendel-Str. 4, 85354 Freising, Germany

**Keywords:** Stachybotryotoxicosis, Sick building syndrome, Indoor air quality, Water damage, Diagnostic test kit, *sat14* gene

## Abstract

Cytotoxic macrocyclic trichothecenes such as satratoxins are produced by chemotype S strains of *Stachybotrys chartarum*. Diseases such as stachybotryotoxicosis in animals and the sick building syndrome as a multifactorial disease complex in humans have been associated with this mold and its toxins. Less toxic non-chemotype S strains of *S. chartarum* are morphologically indistinguishable from chemotype S strains, which results in uncertainties in hazard characterization of isolates. To selectively identify macrocyclic trichothecene producing *S. chartarum* isolates, a set of *sat14* gene-specific primers was designed and applied in a loop-mediated isothermal amplification (LAMP) assay using neutral red for visual signal detection. The assay was highly specific for *S. chartarum* strains of the macrocyclic trichothecene producing chemotype and showed no cross-reaction with non-macrocyclic trichothecene producing *S. chartarum* strains or 152 strains of 131 other fungal species. The assay’s detection limit was 0.635 pg/rxn (picogram per reaction) with a reaction time of 60 min. Its high specificity and sensitivity as well as the cost-saving properties make the new assay an interesting and powerful diagnostic tool for easy and rapid testing.

**Supplementary Information:**

The online version contains supplementary material available at 10.1007/s00216-021-03436-y.

## Introduction

The dematiaceous hyphomycete fungus *Stachybotrys* (*S.*) *chartarum* (Ehrenb.) S. Hughes plays an important role as a potential health hazard for humans [1, 2] and animals [3] due to its production of highly toxic secondary metabolites, known as mycotoxins. Especially, macrocyclic trichothecenes such as satratoxins [4] produced by *S. chartarum* chemotype S strains have a high cytotoxic potential [5]. In horses and other animals, a disease known as stachybotryotoxicosis was described already in 1945 [6, 7]. The disease’s symptoms are catarrh with hemorrhage and ulceration of the mucosae of mouth, nose, and throat, followed by lymphadenopathy with fever, leucopenia, agranulocytic anemia, and death. They result from the ingestion of moldy fodder that had been contaminated with *S. chartarum* and its associated mycotoxins. Moreover, also inhalation of *S. chartarum*-contaminated farm dust and spores were demonstrated to result in clinical signs of stachybotryotoxicosis in animals and farm workers [8]. Skin contact with farm dust and moldy straw bedding resulted in a moist dermatitis with crusts of dried serous exudation in farm animals and workers.

Besides its role in stachybotryotoxicosis, *S. chartarum* was discussed as being involved in fatal cases of pulmonary hemorrhage in infants, which occurred in the 1990s after extensive flooding in Cleveland, Ohio [9, 10]. The toxic fungus was regularly isolated from water damaged cellulosic materials such as wallpapers, plasterboard, or wood [11–14]. Other studies confirmed this correlation [15, 16]. Moreover, *S. chartarum* has been associated with the sick building syndrome [17, 18], a difficult to define medical condition that is caused by multiple factors from the indoor environment of a building. Even though some of the implications that *S. chartarum* might have on humans are under discussion today [19, 20], its negative influence on animal health and the involvement of macrocyclic trichothecenes in its pathology are beyond doubt. The detection of airborne macrocyclic trichothecenes proved that exposure to these toxins is possible by inhalation [21–23]. Furthermore, also foodstuffs such as dried culinary herbs were recently reported to be a possible vector of *Stachybotrys chartarum* [24].

Since *S. chartarum* is a hazardous fungus, the distinction of strains that produce rather harmless atranones from strains that produce highly toxic macrocyclic trichothecenes is of high importance for the estimation of the toxicological potential of animal feed and food as well as for the hazard assessment of indoor environments. The distinction of strains according to chemotypes has been achieved in the past by using liquid chromatography with mass spectrometry analysis for identification of compounds (LC-MS/MS) [25, 26] or through cytotoxicity testing of culture extracts using the MTT assay [27, 28]. However, both methods are complex, expensive, and time-consuming because fungal isolates must be cultivated up to 3 weeks [27] for analytes to be produced. Indirect methods such as micro- and macro-morphology of cultures and matrix-assisted laser desorption/ionization time-of-flight mass spectrometry (MALDI-TOF MS) failed to distinguish highly toxic strains from others [29, 30]. Distinguishing chemotypes also failed with polymerase chain reaction (PCR) assays that were based either on sequences of the 18S rRNA gene [31] or the ITS1-5.8S-ITS2 rRNA gene region [32]. With a C to T exchange in nucleotide position 279 of the *tri5* gene, Andersen et al. distinguished between genotype S and genotype A in strains of *S. chartarum* [26, 33]. However, this distinction was only poorly correlated with the two chemotypes of the species. A recent study has found that the two chemotypes known in *S. chartarum* are represented by three different genotypes instead of two [34]. In contrast to genotypes A and H (both atranone-producing chemotype A), strains of genotype S produce satratoxins F, G, and H and other highly toxic macrocyclic trichothecenes like verrucarin J, roridin E, and L-2 (chemotype S) [35, 36]. It was demonstrated that this genotype exclusively harbors the complete set of 21 genes in the *sat*-cluster that is necessary for the production of macrocyclic trichothecenes. Chemotype A strains were either devoid of *sat*-genes (genotype A) or the gene cluster was incomplete (genotype H). In particular, genes *sat11* through *sat16* were exclusively present in strains that produced macrocyclic trichothecenes in culture [34].

Loop-mediated isothermal amplification (LAMP) is a DNA-based molecular technology that uses a set of four oligonucleotide primers, which need to hybridize to six different locations in the genome of a target organism before enzymatic autocycling in vitro biosynthesis of DNA occurs under isothermal conditions at 65 °C [37, 38]. Advantages of LAMP over PCR-based assays are their higher reaction speed, simplicity of application, and reduced proneness to inhibitors present in sample materials. Its characteristics make LAMP an ideal tool for point of analysis (POA) applications. Due to the application of color change reactions, a visual readout of results can be performed with the naked eye immediately after the reaction has been terminated. Among other organisms, various assays have been designed for the specific detection of filamentous fungi and yeasts [39]. Only recently, the group-specific detection of the *fum1-*gene in fumonisin-producing *Fusarium* spp*.* in maize [40] was successfully demonstrated. Another example for the application of LAMP to the detection of mycotoxin-producing fungi is the detection of patulin producers among *Penicillium* species and its application to the analysis of grapes and apples [41].

The goals of the current study were to set up and optimize a LAMP-based assay for the selective identification of macrocyclic trichothecene producing (genotype S) strains of *S. chartarum* and to determine its characteristics (sensitivity, selectivity).

## Materials and methods

### Chemicals

Tris-HCL and EDTA were purchased from Gerbu Biotechnik GmbH (Heidelberg, Germany). Sodium chloride, sodium acetate, isopropyl alcohol, and dimidium bromide were purchased from Karl Roth GmbH & Co. KG (Karlsruhe, Germany). SDS was purchased from SERVA Electrophoresis GmbH (Heidelberg, Germany). Ethanol analytical grade and acetic acid were purchased from VWR International (Radnor, PA, USA). DNA loading buffer and DNA Ladder GeneRuler 100bp were purchased from Thermo Fisher Scientific Inc. (Waltham, MA, USA). HPLC grade water was used in all experiments unless stated otherwise and was purchased from J.T. Baker (Center Valley, USA).

### Fungal cultures and culture conditions

A complete list of 227 fungal isolates used during the current study is given in Table S1 (see Supplementary Information, ESM). Strains of *S. chartarum* (CBS 414.95, CBS 129.13, and CBS 324.65) were used as reference strains for the genotypes and used for validation and as controls during method development. Cultures were obtained upon direct request to the institutions given as sources. Fungal stock cultures were maintained in glycerol at −80 °C as described by Niessen et al. [42]. Working cultures of all strains were grown on 2.0% malt extract agar plates (MEA, 20 g/L malt extract, 2 g/L soy peptone, and 15 g/L agar (Difco, Heidelberg, Germany), adjust to pH 5.4). Prior to use, all media were sterilized by autoclaving at 121 °C for 15 min. All cultures were grown at ambient temperature (AT, 22 ± 1 °C) for 7 days. Agar cultures were inoculated with a small piece of mycelium from working culture plates and incubated for 5 days at AT. For DNA extraction, cultures were grown in 500 μL of malt extract broth in sterile 1.5 mL reaction vessels at AT on a horizontal shaker at 80 rpm.

### LC-MS/MS measurement

Detection of macrocyclic trichothecenes (satratoxin G, H, and F; roridin E and L-2; verrucarin J) produced by the tested isolates in this study was performed in previous studies by our working group. The LC-MS/MS system consisted of a HPLC device (Shimadzu LC-20AB, SIL-20AC HT, CTO-20AC, CBM-20A, Duisburg, Germany) coupled to an API 4000 triple quadrupole mass spectrometer (Sciex, Darmstadt, Germany). In brief, strains were cultivated on MEA agar for 21 days at 25 °C and plates were extracted by treating in a bag mixer with 50 mL acetonitrile/water 84/16 (v/v). An aliquot (5 mL) of the extract was evaporated to dryness and the residues were reconstituted in 1 mL acetonitrile/water 30/70 (v/v). The limits of detection (LOD) were calculated using the signal-to-noise approach and ranged between 0.1 and 7.8 ng/g MEA agar. For detailed information regarding sample preparation and substance-specific and measurement parameters, refer to Ulrich et al. [34].

### DNA isolation and amplification

DNA extraction from fungal cultures was performed using the protocol described in [43] with some modifications. We used 1.5-mL Eppendorf tubes as culture vessels, containing 500 μL malt extract broth (see above, no agar added). Tubes were inoculated with a loop-full of fungal conidia and incubated horizontally at AT on a rotary shaker at 150 rpm for 2 days. Following centrifugation at 17,900×*g* for 5 min and washing with 500 μL of sterile HPLC grade water, mycelial pellets were mixed with sterile glass beads (1.25–1.65 mm diameter, Carl Roth, Karlsruhe, Germany), sea sand (Merck, Darmstadt, Germany), 300 μL extraction buffer (200 mM Tris-HCl (Gerbu Biotechnik, Heidelberg, Germany), 250 mM sodium chloride (Carl Roth, Karlsruhe, Germany), 25 mM EDTA (Gerbu), 0.5% (w/v) SDS (SERVA Electrophoresis, Heidelberg, Germany), and treated in a TissueLyser (45 s; 5.5 m/s, FastPrep®-24, MP Biomedicals Germany, Eschwege, Germany) to release genomic DNA from cells. The remaining steps were performed as described in Cenis et al. [43]. Vacuum-dried genomic DNA was suspended in sterile HPLC grade water. DNA concentrations were monitored using a NanoDrop ND1000 spectrophotometer (Peqlab Biotechnologie, Erlangen, Germany) according to the manufacturer’s recommendations.

#### PCR amplification of target DNA

Chemotype-specific triplex PCR with DNA of *S. chartarum* isolates was performed using the *Taq* Core Kit 10 (MP Biomedicals) with the primers and cycling protocol described in [34]. The PCR products were separated on 1.3% agarose gel (Biozym Scientific, Hessisch Oldendorf, Germany) at 120 V and 200 mAmp for 1 h and subsequently stained in a dimidium bromide bath for 10 min. Gels were subsequently washed with deionized water for 10 min before visual analysis on a UV transilluminator (UVT-28 M, Herolab, Wiesloch, Germany). PCR products were purified directly from amplification reactions using the QIAquick PCR Purification Kit according to the manufacturer’s instructions (QIAGEN, Hilden, Germany). PCR and LAMP products that were excised from agarose gels with a scalpel were purified using the MinElute Gel Extraction Kit (QIAGEN, Hilden, Germany) according to manufacturer’s instructions.

#### LAMP amplification of target DNA

Primer design was done, using the Primer Explorer V5 online software (Eiken Chemical Co., Ltd., Tokyo, Japan). The master mix for one LAMP reaction contained 2.5 μL 10× ammonium sulfate buffer (100 mM ammonium sulfate (Gerbu), 100 mM potassium chloride (Carl Roth), pH 8.7), 1 μL magnesium chloride (200 mM, Carl Roth), 3.5 μL dNTPs mix (10 mM each GATC, MP Biomedicals GmbH, Eschwege, Germany), 2.6 μL primer mix (1.6 μM each FIP and BIP, 0.8 μM each LF and LB, 0.2 μM each F3 and B3 final concentration in master mix, see Table 1 for primer sequences), 0.75 μL formamide (Sigma-Aldrich, Taufkirchen, Germany), 1 μL *Bst* polymerase (8 U/μL, New England BioLabs, Frankfurt am Main, Germany), 1 μL neutral red (2.5 mM, SERVA Electrophoresis), 7.65 μL sterile deionized UV-treated water, and 5 μL of template DNA per 25 μL of reaction volume. The assay was incubated at 65.5 °C for 60 min in a Mastercycler Gradient Thermal Cycler (Eppendorf, Hamburg, Germany). Genomic DNA of strain *S. chartarum* CBS 414.95 was used as positive control and sterile HPLC grade water was used as negative control throughout the study. Assays for validation were performed in triplicates.

**Table 1:** Oligonucleotides used as primers for LAMP reactions

Nucleotide name	Sequence 5′->3′	Melting temperature (°C)
FIP-SAT 14 ID8	TGTCACACAAGGTGCCCGTC-TCTCAAGTCGAGCGAACTCC*	>75
BIP-SAT 14 ID8	GGTTGAGGTGCCCACTCTCAA-GAACGAATCCATGCCCGG*	>75
F3-SAT 14 ID8	GTTTTCACAGACGCCATCCA	57.3
B3-SAT 14 ID8	TCCCGTCCAATTCCAGTCT	56.7
LF-SAT 14 ID8	CGCACCATTGTTTGAGTCGG	59.4
LB-SAT 14 ID8	CAAGCCTGGTTGGTTGTATATGC	60.6
F2-SAT 14 ID8	TCTCAAGTCGAGCGAACTCC	59.4
B2-SAT 14 ID8	GAACGAATCCATGCCCGG	58.2

*Hyphenation indicates interface between F1c/B1 and F2/B2c parts of composite primers

A few reactions were performed with the ESEQuant TS real-time fluorimeter (QIAGEN Lake Constance GmbH, Stockach, Germany) to determine the effect of our loop primers on the reaction. V13 (V13-01184, Dyomics GmbH, Jena, Germany) was used as a fluorescent indicator dye instead of neutral red using all other reaction components as previously described.

#### Assay validation

Cross-reactions in the assay were eliminated by increasing formamide concentration (see Fig. S2 in ESM) and temperature (see Fig. S1 in ESM). The presence of the correct amplified LAMP product in positive LAMP reactions was confirmed by sequence analysis of the smallest DNA fragment visible when LAMP reactions were separated by agarose gel electrophoresis. The smallest amplified product was cut out from the gel and a PCR with the primers F2 and B2 (part of FIP and BIP, see Table 1) was done with the purified DNA fragment as template. Subsequently, the PCR product was isolated as described previously and Sanger sequenced using primers F2 and B2. Sequences were pairwise aligned and a consensus sequence was generated.

The effect of loop primers LF and LB (see Table 1) on the reaction speed is a helpful parameter for the fine-tuning of a LAMP assay. We measured this effect in a real-time ESE Quant TS tube scanner fluorimeter (QIAGEN Lake Constance, Germany) using the V 13-01184 fluorescent dye (Dyomics, Jena, Germany) as indicator.

The sensitivity of the assay was analyzed by testing a serial dilution of *S. chartarum* CBS 414.95 gDNA as template.

## Results

### Development and optimization of the LAMP assay

A set of LAMP primers, including loop primers, were designed using the Primer Explorer V5 software. Since *S. chartarum* genotypes A and H, as well as *S. chlorohalonata*, lack the presence of the satratoxin core cluster 2 (SC2) [34, 44], we designed our primer set (see Table 1) to hybridize with the *sat14* gene exclusively present in SC2 of genotype S strains of *S. chartarum* (= chemotype S). If a strain belongs to chemotype S, the primers hybridize with the *sat14* gene and DNA is amplified during the LAMP reaction. Positive results are visualized by a color change from yellow to pink and can be read with the naked eye due to the utilization of neutral red as pH indicator. Cross-reactions in our LAMP were eliminated step by step by adding formamide to the master mix and increasing the reaction temperature, thus making the reaction more specific. At low temperatures and without the addition of formamide to the reaction, the binding of the primers became unspecific which led to cross-reactions with several of the tested fungal species. Highly specific reactions occurred however, when 0.75 μL of formamide was added per reaction. Optimization experiments using the temperature gradient function of the thermal cycler revealed 65.5 °C with a reaction time of 60 min as optimum reaction conditions for the rapid, sensitive, and specific amplification of target DNA.

**Fig. 1:**
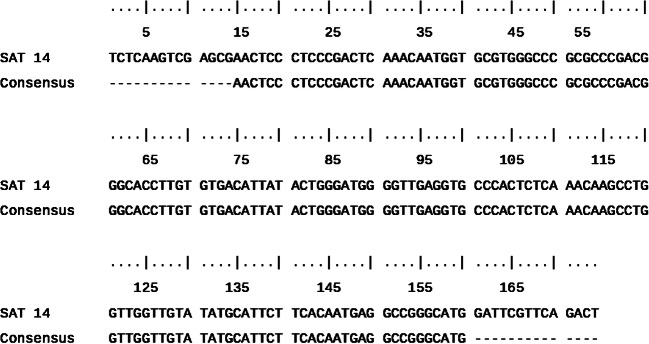
Comparison of the *sat14* gene partial sequence with a consensus sequence of the PCR product obtained with primers F2/B2 on the smallest DNA fragment produced during reactions with the *sat14* gene specific LAMP assay

The alignment of the obtained sequence with the *sat14* gene sequence is shown in Fig. 1. Results showed a 100% homology between both sequences confirming the correct binding and specificity of the designed primers.

**Fig. 2:**
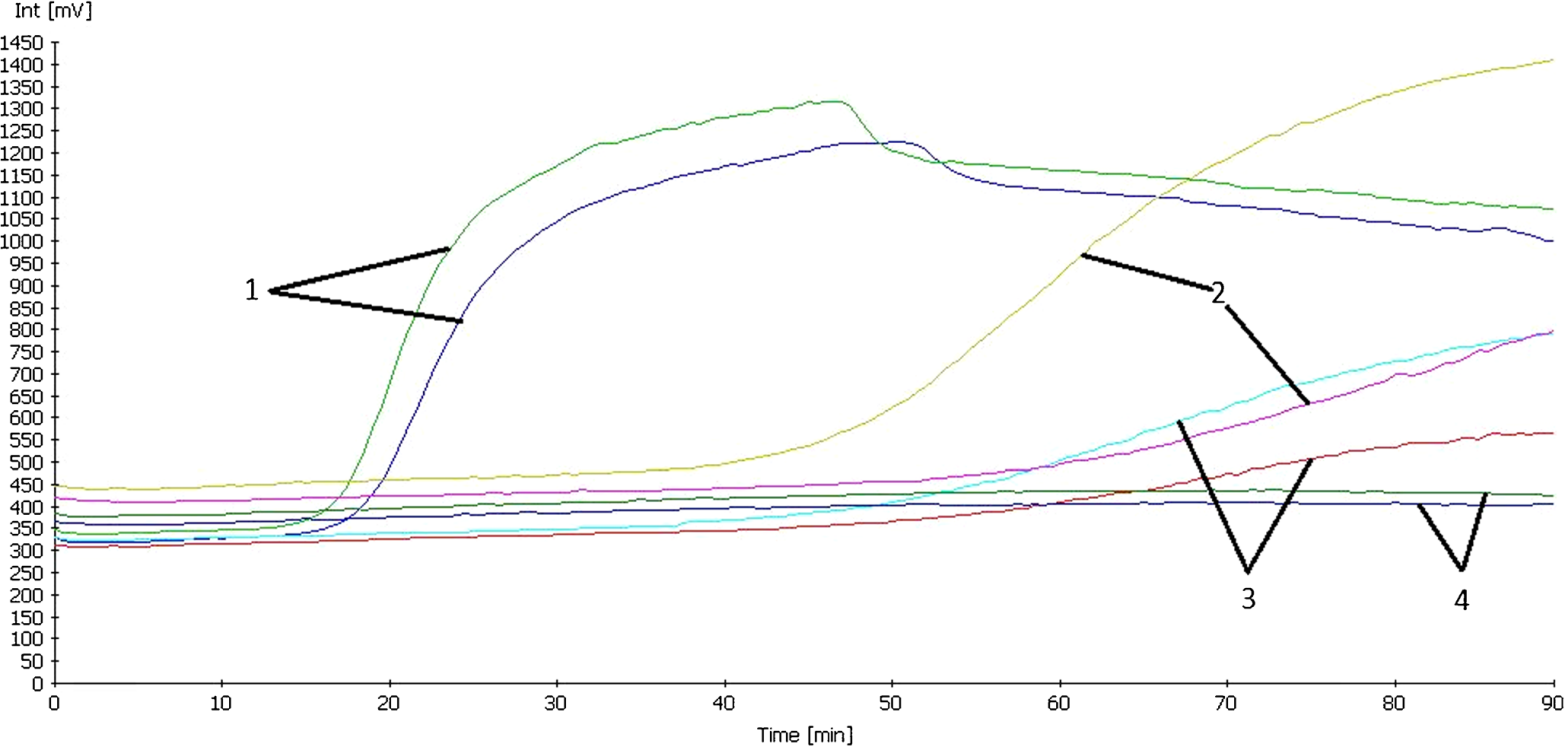
Influence of loop primers on reaction speed of the *sat14* gene specific LAMP reaction using V13 as fluorescent indicator. Graph-pair 1: Both LF + LB loop primers added to the reaction. Graph-pair 2: only LB primer added to the reaction. Graph-pair 3: only LF primer added to the reaction. Graph-pair 4: no loop primers added to the reaction

Figure 2 shows the influence of both loop primers on the reaction speed. Using purified gDNA of *S. chartarum* CBS 414.95 as template, the LAMP reaction showed no measurable signal within 90 min runtime when none of the loop primers LF or LB was added. Addition of either of primers LF or LB resulted in a LAMP signal starting at about 50 min runtime. A fluorescent signal that emerged from the background after only 20 min was detected in the LAMP reaction when both primers LF and LB were added to the master mix.

**Fig. 3:**
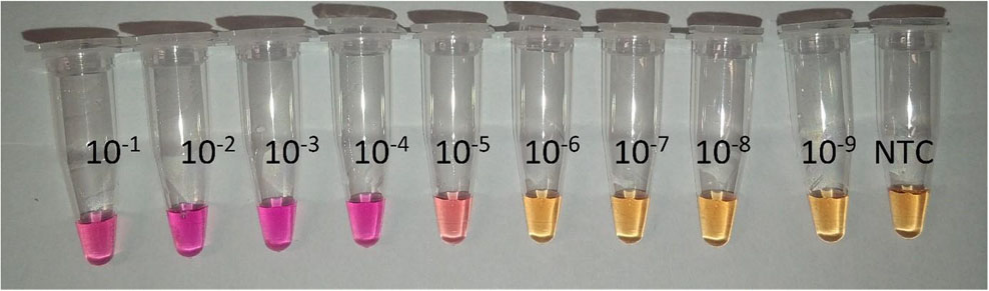
*Sat14* gene specific LAMP reaction with a serial dilution of gDNA of reference strain CBS 414.95 using neutral red as pH sensitive indicator (positive reaction indicated by color change from yellow to purple). Cap with 10 ^-1^ holds 1.27 ng/μl (equivalent to 6.35 ng per reaction) as initial template DNA concentration, NTC = No template control

The sensitivity of the optimized assay was analyzed by the addition of purified gDNA from *S. chartarum* CBS 414.95 as template from a 10-fold serial dilution. Figure 3 shows that the addition of template DNA from a 10^−5^ dilution still resulted in a visible color change when neutral red was used as an indicator that changes color from yellow to pink when the pH drops during positive LAMP reactions. This dilution was equivalent to a concentration of 6.35 pg template DNA per reaction. *S. chartarum* has a haploid genome size of 36.5 Mbp. Therefore, the limit of detection of the LAMP assay translates into a genomic copy number of 15.8 cp/reaction. Since the *sat14* gene is a single-copy gene, the assay can detect a limit of 15.8 theoretical cells per reaction [44].

During the current study, a total of 227 fungal isolates were analyzed using the newly developed LAMP assay (see Table S1 in ESM). The tested isolates included 75 strains of *S. chartarum* (see Table 2). Among the tested *S. chartarum* strains, 30 genotype S, 29 genotype A, and 14 genotype H were identified by triplex PCR according to [34]. Three strains (CBS 414.95, CBS 129.13, and CBS 324.65) were used as reference strains for the genotypes. Two strains (S1344, IBT 8935) could not be assigned to any of the genotypes using triplex PCR but reacted positive in the LAMP assay. LAMP with all *S. chartarum* genotype S strains resulted in a positive signal, whereas all strains of genotypes A and H showed negative results in the LAMP assay. Also included in the total of the tested strains were 29 strains of other *Stachybotrys* species including 21 strains of the closely related *S. chlorohalonata* as well as one strain each of eight other *Stachybotrys* species and two strains of *Memnoniella echinata* as a more remotely related species (for all *S. chartarum* see ESM Table S1). None of the tested strains reacted positive in the LAMP assay. In order to test unrelated species, 121 isolates of fungal species were tested that potentially occur in environments in which *S. chartarum* can be found (plant material, indoor environments, straw bedding). All isolates tested negative with the gene-specific LAMP assay.

**Table 2:** Strains of *S. chartarum* (n = 75) with *sat*-genotype and result in the genotype S-specific LAMP reaction

Genus	Species	Strain ID	Source	*sat*-genotype1	LAMP result^2^
*Stachybotrys*	*chartarum*	S 1352	LMU^3^	S	+^4^
	*chartarum*	S 1344	LMU	n.r.^5^	+
	*chartarum*	S 1349	LMU	S	+
	*chartarum*	S 1418/1	LMU	S	+
	*chartarum*	S BB2	LMU	S	+
	*chartarum*	S BO1a	LMU	S	+
	*chartarum*	R07	LMU	S	+
	*chartarum*	R06	LMU	S	+
	*chartarum*	S 1425	LMU	S	+
	*chartarum*	S BT3	LMU	S	+
	*chartarum*	S BO2	LMU	S	+
	*chartarum*	S BO1b	LMU	S	+
	*chartarum*	CBS^6^ 414.95	TUM^7^	S	+
	*chartarum*	R 24	LMU	S	+
	*chartarum*	S 1114	LMU	S	+
	*chartarum*	S 1493/1	LMU	S	+
	*chartarum*	S 1166/2	LMU	S	+
	*chartarum*	S 1455	LMU	S	+
	*chartarum*	S 1492	LMU	S	+
	*chartarum*	S 4	LMU	S	+
	*chartarum*	S 5	LMU	S	+
	*chartarum*	S 9	LMU	S	+
	*chartarum*	S 16St	LMU	S	+
	*chartarum*	S 23St	LMU	S	+
	*chartarum*	S 35It	LMU	S	+
	*chartarum*	S 48St	LMU	S	+
	*chartarum*	H 47A	LMU	S	+
	*chartarum*	H 47D	LMU	S	+
	*chartarum*	IBT^8^ 7709	IBT	S	+
	*chartarum*	TMW_4.689	TUM	S	+
	*chartarum*	IBT 8935	IBT	n.r.	+
	*chartarum*	Sp 2675	TUM	S	+
	*chartarum*	S 1433	LMU	A	-^9^
	*chartarum*	S 1432	LMU	A	-
	*chartarum*	S 1431	LMU	A	-
	*chartarum*	S 1409	LMU	A	-
	*chartarum*	S 1378	LMU	A	-
	*chartarum*	S 1362	LMU	A	-
	*chartarum*	S 1353	LMU	A	-
	*chartarum*	S 1432	LMU	A	-
	*chartarum*	S 1074	LMU	A	-
	*chartarum*	S 1286	LMU	A	-
	*chartarum*	S 1387	LMU	A	-
	*chartarum*	S 1362	LMU	A	-
	*chartarum*	S 1091	LMU	A	-
	*chartarum*	S 1494	LMU	A	-
	*chartarum*	S 24It/B	LMU	A	-
	*chartarum*	IBT 8709	IBT	A	-
	*chartarum*	TMW_4.685	TUM	A	-
	*chartarum*	TMW_4.674	TUM	A	-
	*chartarum*	S 1433	LMU	A	-
	*chartarum*	CBS 129.13	TUM	A	-
	*chartarum*	S 1244	LMU	A	-
	*chartarum*	TMW_4.675	TUM	A	-
	*chartarum*	S 1589	LMU	A	-
	*chartarum*	TMW_4.678	TUM	A	-
	*chartarum*	TMW_4.680	TUM	A	-
	*chartarum*	TMW_4.682	TUM	A	-
	*chartarum*	TMW_4.684	TUM	A	-
	*chartarum*	R10	LMU	A	-
	*chartarum*	R09	LMU	A	-
	*chartarum*	S6OW	LMU	H	-
	*chartarum*	S 1285	LMU	H	-
	*chartarum*	S 41	LMU	H	-
	*chartarum*	S 42	LMU	H	-
	*chartarum*	S 43	LMU	H	-
	*chartarum*	S 1341	LMU	H	-
	*chartarum*	S 1342	LMU	H	-
	*chartarum*	S 1335	LMU	H	-
	*chartarum*	S 3	LMU	H	-
	*chartarum*	CBS 324.65	TUM	H	-
	*chartarum*	S 1077	LMU	H	-
	*chartarum*	S 1333	LMU	H	-
	*chartarum*	S 1334	LMU	H	-
	*chartarum*	S 1339	LMU	H	-

^1^According to Ulrich et al. [34]

^2^According to the current study

^3^Strain collection at Chair of Food Safety, Ludwig-Maximilian-University, Munich, Germany

^4^Positive result

^5^No result

^6^CBS strain collection at Westerdijk Fungal Biodiversity Institute, Utrecht, The Netherlands

^7^Chair of Technical Microbiology, School of Life Sciences Weihenstephan, Technical University of Munich, Germany

^8^Culture Collection of Fungi, Danish Technical University, Lyngby, Denmark

^9^Negative result

Every isolate of the 30 *S. chartarum* Chemotype S which all had a positive result in the *sat14*-specific LAMP reaction showed the production of macrocyclic trichothecenes in culture as measured by LC-MS/MS in previous studies [24, 34, 45, 46]. Table 3 shows the isolates with underlying data for LC-MS/MS compared to LAMP results. Genotypes A and H, as well as closely related *Stachybotrys* species, neither showed a positive reaction in the new LAMP assay nor did they produce any of the analyzed macrocyclic trichothecenes. As an exception, the isolate *S. dichroa* ATCC 18913 was reported to produce roridin E [36] and verrucarin J [46], but lacks the ability to produce satratoxins. However, it correctly proved negative in the LAMP assay.

**Table 3:** Comparison of selected isolates with data on macrocyclic trichothecene production as determined by LC-MS/MS and result in the genotype S specific LAMP reaction

Genus	Species	Strain ID	Source	*sat*-genotype1	LAMP result2	Toxin production (LC-MS/MS)3
*Memnoniella*	*echinata*	MYA 584	LMU^4^	n.a.^5^	-^6^	n.d.^7^
*Stachybotrys*	*chartarum*	S BB2	LMU	S	+^8^	+
	*chartarum*	S BO1a	LMU	S	+	+
	*chartarum*	S BT3	LMU	S	+	+
	*chartarum*	S BO2	LMU	S	+	+
	*chartarum*	S BO1b	LMU	S	+	+
	*chartarum*	S 1114	LMU	S	+	+
	*chartarum*	S 16St	LMU	S	+	+
	*chartarum*	S 35It	LMU	S	+	+
	*chartarum*	H 47D	LMU	S	+	+
	*chartarum*	S 1166/2	LMU	S	+	+
	*chartarum*	S 1455	LMU	S	+	+
	*chartarum*	S 1492	LMU	S	+	+
	*chartarum*	S 5	LMU	S	+	+
	*chartarum*	S 23St	LMU	S	+	+
	*chartarum*	H 47A	LMU	S	+	+
	*chartarum*	S 4	LMU	S	+	+
	*chartarum*	S 9	LMU	S	+	+
	*chartarum*	S 48St	LMU	S	+	+
	*chartarum*	S 1493/1	LMU	S	+	+
	*chartarum*	CBS^9^ 414.95	TUM^10^	S	+	+
	*chartarum*	Sp 2675	TUM	S	+	+
	*chartarum*	R 24	LMU	S	+	+
	*chartarum*	CBS 129.13	TUM	A	-	n.d.
	*chartarum*	S 1244	LMU	A	-	n.d.
	*chartarum*	S 1074	LMU	A	-	n.d.
	*chartarum*	S 1286	LMU	A	-	n.d.
	*chartarum*	S 1091	LMU	A	-	n.d.
	*chartarum*	S 1494	LMU	A	-	n.d.
	*chartarum*	S 24It/B	LMU	A	-	n.d.
	*chartarum*	S 1589	LMU	A	-	n.d.
	*chartarum*	S 1378	LMU	A	-	n.d.
	*chartarum*	S 1353	LMU	A	-	n.d.
	*chartarum*	S 1432	LMU	A	-	n.d.
	*chartarum*	S 1362	LMU	A	-	n.d.
	*chartarum*	S 1433	LMU	A	-	n.d.
	*chartarum*	S 1431	LMU	A	-	n.d.
	*chartarum*	S 1285	LMU	H	-	n.d.
	*chartarum*	S 1341	LMU	H	-	n.d.
	*chartarum*	S 1342	LMU	H	-	n.d.
	*chartarum*	S 1335	LMU	H	-	n.d.
	*chartarum*	S 3	LMU	H	-	n.d.
	*chartarum*	CBS 324.65	TUM	H	-	n.d.
	*chartarum*	S 1339	LMU	H	-	n.d.
	albipes*	ATCC^11^ 18873	ATCC	n.a.	-	n.d.
	*chlorohalonata*	IBT^12^ 40285	IBT	n.a.	-	n.d.
	*cylindrospora*	ATCC 16276	ATCC	n.a.	-	n.d.
	*dichroa*	ATCC 18913	ATCC	n.a.	-	+
	*kampalensis*	ATCC 22705	ATCC	n.a.	-	n.d.
	*oenanthes*	ATCC 22844	ATCC	n.a.	-	n.d.

*Anamorphic state of *Melanopsamma pomiformis*

^1^According to Ulrich et al. [34]

^2^According to the current study

^3^Detection of macrocyclic trichothecenes according to [24, 34, 45, 46]

^4^Strain collection at Chair of Food Safety, Ludwig-Maximilian-University, Munich, Germany

^5^Not applicable

^6^Negative result

^7^No macrocyclic trichothecenes (satratoxin G, H, and F; roridin E and L-2; verrucarin J) detected by LC-MS/MS (LOD 0.1–7.8 ng/g MEA agar)

^8^Positive result in LAMP/macrocyclic trichothecenes detected by LC-MS/MS

^9^CBS strain collection at Westerdijk Fungal Biodiversity Institute, Utrecht, The Netherlands

^10^Chair of Technical Microbiology, School of Life Sciences Weihenstephan, Technical University of Munich, Germany

^11^ATCC American Type Culture Collection, Manassas, USA

^12^Culture Collection of Fungi, Danish Technical University, Lyngby, Denmark

Specificity testing performed during the current study demonstrated clearly that the designed set of LAMP primers selectively bind to the DNA of genotype S strains of *S. chartarum*. No cross-reactions were detected with non-satratoxin-producing strains (genotypes A and H) or with closely related *Stachybotrys* species. Also, the various other tested fungal species did not result in positive LAMP reactions. In order to reach these results, the assay conditions needed to be optimized regarding incubation temperature and the addition of formamide to the master mix to reduce cross-reactions. Moreover, adding an additional pair of loop primers resulted in a considerable reduction of the reaction time as compared to the reaction time without loop primers.

## Discussion

According to Semeiks et al. [44], 21 genes are necessary in *S. chartarum* to produce satratoxins. Previous studies showed that only those strains of the fungus are able to produce satratoxins that harbor the full set of genes in their genomes, including genes *sat11* through *sat16* (the satratoxin core cluster 2 (SC2) genes) [34]. Moreover, it was demonstrated that non-satratoxin-producing strains of *S. chartarum* could have two different genotypes, which either have none of the *sat*-genes or miss *sat11* through *sat16* (the SC2). In some genotype H strains, also some of the genes in SC1 and SC3 seemed to be missing or truncated [34]. In order to differentiate satratoxin-producing strains from non-satratoxin-producing strains of *S. chartarum*, we based our assay on the detection of sequences from one of the genes in SC2. Comparing *sat*-gene sequences of two strains of *S. chartarum* for which annotated genomic sequences are available, we found that the *sat14* gene showed the lowest number of mismatches and SNPs. Moreover, the gene had the lowest number of introns among the six genes in SC2. Both features present in high numbers would bear a potential of designing primers that will not properly hybridize to the DNA of all strains of the target species.

As we pointed out earlier, the LAMP reaction is characterized by a high level of sensitivity so that even small amounts of DNA lead to a positive result. On the other hand, too high DNA concentrations can inhibit the amplification and may lead to false negative results. Thus, measuring concentrations and diluting samples correctly are important. Particular care should be taken with false negative samples when testing on site, as no purified DNA sample is tested and therefore a reliable measurement of DNA concentration as well as occurring reaction inhibitors in the sample are problems to be dealt with. With an adequate adjustment of reaction temperature and formamide concentration, false positive reactions were completely excluded and did not occur among the tested isolates in this study.

The isolate *S. dichroa* ATCC 18913 revealed a negative result in the *sat14*-specific LAMP reaction. However, it was previously shown that this isolate produced the macrocyclic trichothecenes roridin E [36] and verrucarin J [46], but no satratoxins. Our LAMP reaction amplifies the *sat14* gene which the fungus needs to synthesize this specific group of toxins and that in turn corresponds to an absence of *sat14*. This observation highlights ambiguities in the metabolite profile of *Stachybotrys* species, which is still part of ongoing research [47, 48].

During the current study, we used neutral red as a pH-sensitive indicator for in-tube indirect visual signal detection under daylight conditions. The advantage of this indicator compared to turbidimetric or fluorescence-based detection of LAMP signals [49] is that results can be read visually without the need for a device such as a turbidimeter, fluorimeter, or even a UV lamp. Moreover, neutral red can be added to the master mix before the reaction starts; it is not toxic for those handling it [50] and does not interfere with the LAMP reaction. This indicator was used successfully in several previous studies [40, 51, 52]. Furthermore, a big advantage is the minimization of possible lab space contamination with product DNA. This is because a color change indicates a positive reaction, and no further handling of the DNA, like gel electrophoresis, is needed. Optimization and specificity testing were performed using genomic DNA after isolation from pure culture mycelia. As was reported in literature, LAMP assays can also be successfully performed using direct addition of fungal spores as template [42, 53–55]. However, during the current study, it turned out that direct addition of spore material of *S. chartarum* genotype S strains into the master mix did not lead to reliable results (results not shown). Mechanical disruption of the spores by vortexing with and without glass beads, as well as thermal treatment, did not result in the release of sufficiently reproducible gDNA amounts that are needed for a reproducible output of the LAMP assay. We assume that factors such as melanin [29] and polysaccharides from the cell wall and proteinases from the cytoplasm might inhibit the polymerase used in the LAMP reaction. Moreover, DNAses might lead to the rapid digestion of the genomic DNA, hampering its detection by the assay. A working on-site application could contain a portable DNA extraction kit and a portable battery-operated heating device like the ESEQuant TS real-time fluorimeter (QIAGEN Lake Constance GmbH, Stockach, Germany), which was used in this study. As already mentioned, experiments with direct measurement of spores and also with a strongly simplified DNA extraction were not successful by our working group, but we suggest experimental approaches using glass beads/sea sand or similar disruption media combined with vortexing and/or thermal disruption to get usable amounts of DNA for LAMP. Thermal treatment was used successfully by Jayanath et al. in detecting hepatitis B virus in human serum by LAMP [56]. However, according to our experience, it takes higher efforts to disrupt the rigid cell walls of fungal spores. Another study showed that human serum has an inhibitory effect on LAMP reactions [57]. Also, this observation coincides with our experience of inhibitory fungal cell components such as DNAses. An approach to exclude such components from extracts could be a syringe filter-based DNA extraction like the one described by Lee et al. [58]. The most simple way of LAMP analysis of fungi is the direct addition of spores or mycelia into the LAMP master mix as demonstrated by several studies [42, 53, 55, 59]. All chemicals that we used in the current LAMP assay can be transported on-site, e.g., in an ice box. For long time storage of LAMP reagents without cooling, freeze drying of the master mix might be an alternative. However, further research is needed here.

Macrocyclic trichothecenes such as satratoxins are hazardous to the health of farm animals and humans living or working in moldy environments. To detect these toxins in feed, food, or environmental samples, contaminated materials can be analyzed by LC-MS/MS, ELISA, and MTT tests [21–23, 27, 28, 60]. Such tests are more or less expensive and time-consuming because samples have to be sent to a testing facility with special instrumentation, while LAMP is a format that involves little costs, time, and equipment, and could potentially be applied even in the field [61, 62]. Therefore, the assay developed during the current study may pave the way for the development of rapid test kits for animal feed and bedding materials and for testing of indoor environments in human dwellings and workplaces. For the implication of these tests to field conditions, further research is needed to set up sample preparation protocols for the rapid direct analysis of sample materials.

## Supplementary Information

ESM 1 (PDF 485 kb)

## Data Availability

All methods and materials have been described in full. All used cultures are available from public strain collections as indicated in the text or are available from the authors’ in house collection on request.
