# Supplementary material for: Rapid and selective detection of macrocyclic trichothecene producing Stachybotrys chartarum strains by loop-mediated isothermal amplification (LAMP)
Source: Anal Bioanal Chem. 2021 Jun 15;413(19):4801–13. doi: 10.1007/s00216-021-03436-y (PMC8318954; doi:10.1007/s00216-021-03436-y)
Supplement: Supplementary file 1 — (PDF 485 kb) [file 216_2021_3436_MOESM1_ESM.pdf]

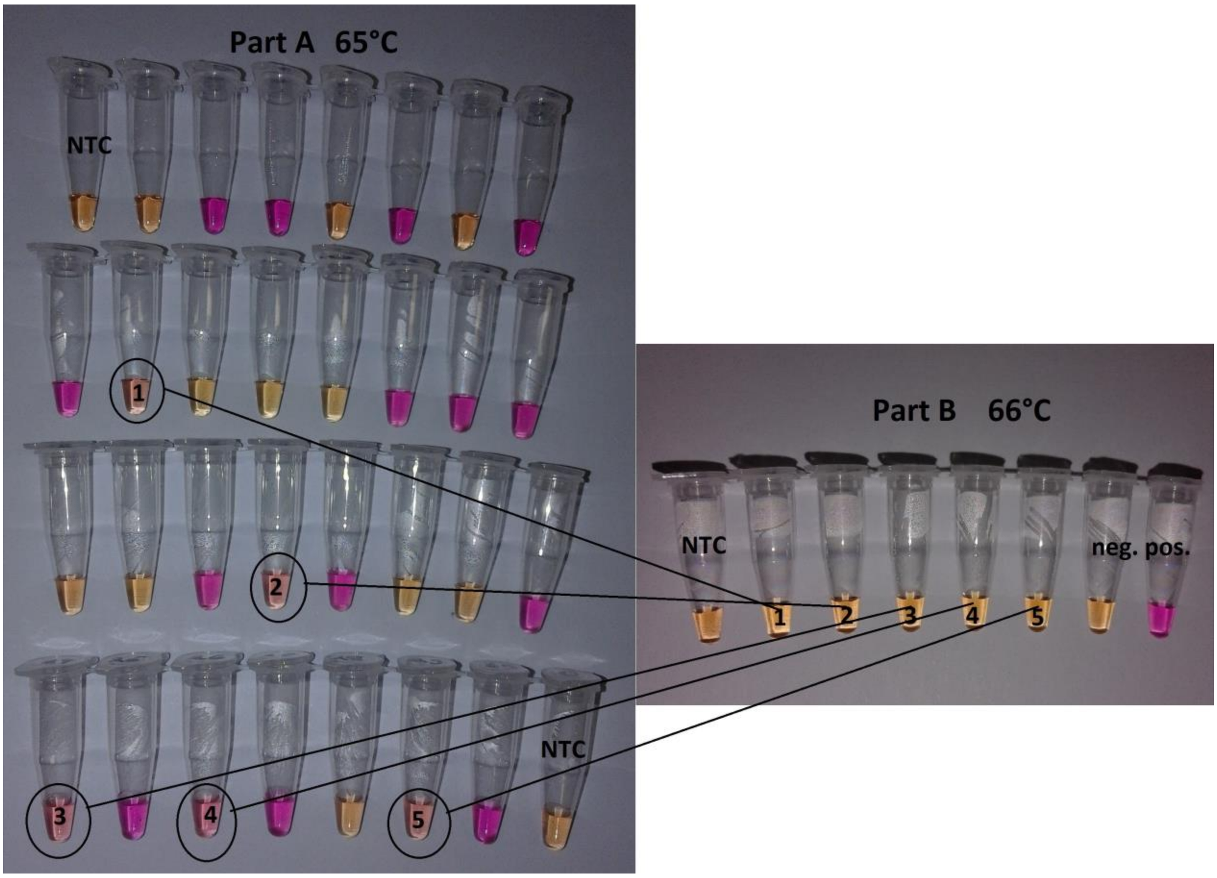

Figure S1: Influence of incubation temperature on the specificity of the LAMP reaction. A: LAMP reactions with DNA of S. chartarum genotypes S, A and H as well as S. chlorohalonata at 65°C for one hour. Pink reaction vessels contain DNA from S. chartarum genotype S as template. Yellow coloured vessels contain DNA from other genotypes and show a negative result. Reactions marked 1-5 are false positives (1: S. chartarum genotype A, 2: S. chartarum genotype H, 3: S. chlorohalonata, 4: S. chlorohalonata, 5: S. chlorohalonata). NTC: No template control.
B: LAMP reactions with template DNA used in reactions marked 1-5 after one hour of incubation at 66 °C. Note that the reactions are clearly negative under the optimized conditions. Neg.: negative control S. chartarum genotype A, pos.: positive control S. chartarum genotype S, NTC: No template control.

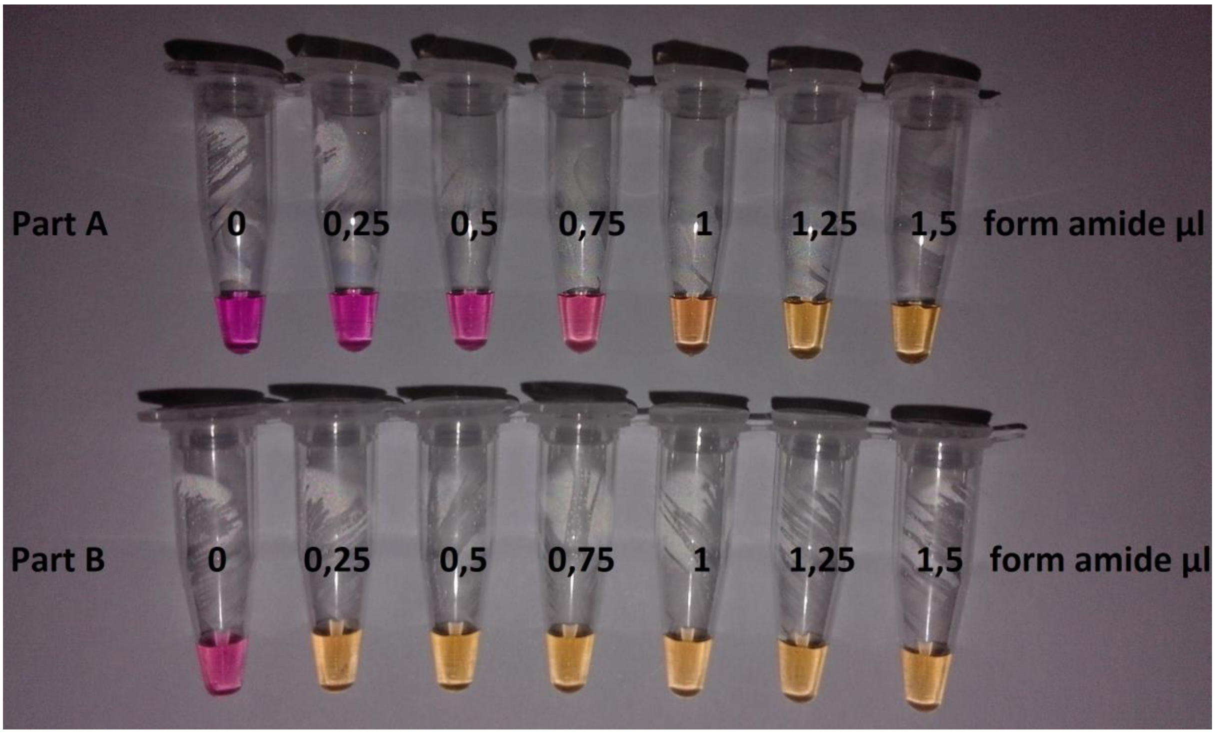

Figure S2: Influence of form amide on the specificity of the LAMP reaction. A: LAMP reactions with DNA from S. chartarum genotype S as template and addition of 0.0 to 1.5 µL form amide per reaction. Note that addition of 0.75 µL per reaction still results in a positive LAMP reaction within one hour of incubation. Higher amounts of formamide inhibit the reaction. B: LAMP reaction with DNA from S. chlorohalonata as template and addition of 0.0 to 1.5 µL form amide per reaction. Note that the reaction was positive with 0.0 µL form amide per reaction. Addition of 0.25 to 1.5 µL per reaction lead to complete inhibition of the LAMP reaction

Table S1: List of fungal species and strains (n = 227) used in the current study with result obtained with the newly developed LAMP assay.

Genus	Species	Strain ID	Source	*sat*-genotype[1]	LAMP-result[2]	Toxin production (LC-MS/MS)[3]
Alternaria	*alternata*	TMW 4.0438	TUM[4]	n.a.[5]	-[6]	n.a.
	mali	CBS 106.24	CBS[7]	n.a.	-	n.a.
Aspergillus	*aculeatus*	TMW 4.1776	TUM	n.a.	-	n.a.
	alliaceus	DSM 813	DSM[8]	n.a.	-	n.a.
	arachidicola	IBT 27128	IBT[9]	n.a.	-	n.a.
	auricomus	CBS 467.65	CBS	n.a.	-	n.a.
	awamori	CBS 101704	CBS	n.a.	-	n.a.
	bombycis	IBT 23536	IBT	n.a.	-	n.a.
	bridgeri	CBS 350.81	CBS	n.a.	-	n.a.
	caelatus	IBT 29700	IBT	n.a.	-	n.a.
	carbonarius	TMW 4.1512	TUM	n.a.	-	n.a.
	clavatus	CBS 513.65	CBS	n.a.	-	n.a.
	elegans	CBS 310.80	CBS	n.a.	-	n.a.
	ellipticus	CBS 707.79	CBS	n.a.	-	n.a.
	flavus	TMW 4.1859	TUM	n.a.	-	n.a.
	foetidus	CBS114.49	CBS	n.a.	-	n.a.
	fresenii	CBS 550.65	CBS	n.a.	-	n.a.
	fumigatus	CBS 113.55	CBS	n.a.	-	n.a.
	helicothrix	CBS 677.79	CBS	n.a.	-	n.a.
	heteromorphus	CBS 117.55	CBS	n.a.	-	n.a.
	insulicola	CBS 382.75	CBS	n.a.	-	n.a.
	japonicus	CBS 114.51	CBS	n.a.	-	n.a.
	minisclerotigenes	IBT 27177	IBT	n.a.	-	n.a.
	niger	CBS 101698	CBS	n.a.	-	n.a.
	nomius	CBS 260.88	CBS	n.a.	-	n.a.
	ochraceoroseus	CBS 101887	CBS	n.a.	-	n.a.
	ochraceus	CBS 263.67	CBS	n.a.	-	n.a.
	oryzae	IBT 28103	IBT	n.a.	-	n.a.
	parasiticus	CBS 126.62	CBS	n.a.	-	n.a.
	parvisclerotigenes	IBT 3850	IBT	n.a.	-	n.a.
	petrakii	CBS 105.57	CBS	n.a.	-	n.a.
	pseudotararii	IBT 21092	IBT	n.a.	-	n.a.
	rambellii	IBT 14580	IBT	n.a.	-	n.a.
	sclerotiorum	CBS 549.65	CBS	n.a.	-	n.a.
	sojae	IBT 21643	IBT	n.a.	-	n.a.
	tamarii	CBS 591.68	CBS	n.a.	-	n.a.
	terreus	CBS 377.64	CBS	n.a.	-	n.a.
	toxicarius	CBS 822.72	CBS	n.a.	-	n.a.
	tubingensis	ITEM 4496	ITEM[10]	n.a.	-	n.a.
	usamii	CBS 101700	CBS	n.a.	-	n.a.
Aureobasidium	*pullulans*	TMW 4.2253	TUM	n.a.	-	n.a.
Beltraniella	*portoricensis*	CBS 856.70	CBS	n.a.	-	n.a.
Bipolaris	*sorokiniana*	CBS 311.64	CBS	n.a.	-	n.a.
Cladobotryum	*dendroides*	NRRL 2903	NRRL[11]	n.a.	-	n.a.

Cladosporium	*sphaerospermum*	TMW 4.2370	TUM	n.a.	-	n.a.
Colletotrichum	*acutatum*	CBS 295.67	CBS	n.a.	-	n.a.
	fragariae	CBS 142.31	CBS	n.a.	-	n.a.
	gloeosporioides	CBS 285.50	CBS	n.a.	-	n.a.
Cryptomela	*acutispora*	CBS 157.33	CBS	n.a.	-	n.a.
Drechslera	*teres*	CBS 378.59	CBS	n.a.	-	n.a.
	tricici-repentis	CBS 265.80	CBS	n.a.	-	n.a.
Emericella	*astellata*	IBT 21903	IBT	n.a.	-	n.a.
	olivicola	IBT 26499	IBT	n.a.	-	n.a.
	venezuelensis	IBT 20956	IBT	n.a.	-	n.a.
Epicoccum	*nigrum*	TMW 4.1407	TUM	n.a.	-	n.a.
Fusarium	*acuminatum*	CBS 485.94	VBS	n.a.	-	n.a.
	avenaceum	DSM 62161	DSM	n.a.	-	n.a.
	beomiforme	BBA 69406	BBA[12]	n.a.	-	n.a.
	cerealis	CBS 589.93	CBS	n.a.	-	n.a.
	chlamydosporum	CBS 145.25	CBS	n.a.	-	n.a.
	compactum	CBS 466.92	CBS	n.a.	-	n.a.
	culmorum	DSM 62191	DSM	n.a.	-	n.a.
	dimerum	CBS 175.31	CBS	n.a.	-	n.a.
	dlaminii	MRC 3024	MRC[13]	n.a.	-	n.a.
	equiseti	CBS 406.86	CBS	n.a.	-	n.a.
	eumartii	DSM 62809	DSM	n.a.	-	n.a.
	heterosporum	DSM 62231	DSM	n.a.	-	n.a.
	longipes	CBS 739.79	CBS	n.a.	-	n.a.
	melanochlorum	CBS 202.65	CBS	n.a.	-	n.a.
	napiforme	BBA 67629	BBA	n.a.	-	n.a.
	oxysporum	DSM 62292	DSM	n.a.	-	n.a.
	proliferatum	DSM 62261	DSM	n.a.	-	n.a.
	scirpi	CBS 448.84	CBS	n.a.	-	n.a.
	solani	DSM 62416	DSM	n.a.	-	n.a.
	subglutinans	BBA 63621	BBA	n.a.	-	n.a.
	sublunatum var.	CBS 189.34	CBS	n.a.	-	n.a.
	torulosum	BBA 64465	BBA	n.a.	-	n.a.
Geomyces	*auratus*	BBA 66636	BBA	n.a.	-	n.a.
Geotrichum	*candidum*	TMW 4.0508	TUM	n.a.	-	n.a.
Gliocephalotrichum	*spec. nov.*	NRRL 2993	NRRL	n.a.	-	n.a.
Hypomyces	*rosellus*	CBS 521.81	CBS	n.a.	-	n.a.
Memnoniella	*echinata*	CBS 627.61	CBS	n.a.	-	n.a.
	echinata	MYA 584	LMU	n.a.	-	n.d.
Microdochium	*majus*	TMW 4.0496	TUM	n.a.	-	n.a.
	nivale	TMW 4.0495	TUM	n.a.	-	n.a.
Monascus	*ruber*	TMW 4.1426	TUM	n.a.	-	n.a.
Mucor	*mucedo*	DSM 809	DSM	n.a.	-	n.a.
Myrothecium	*roridum*	CBS 331.51	CBS	n.a.	-	n.a.
Penicillium	*aurantiogriseum*	CBS 225.90	CBS	n.a.	-	n.a.
	brevicompactum	TMW 4.2279	TUM	n.a.	-	n.a.
	camembertii	DSM 1233	DSM	n.a.	-	n.a.
	chrysogenum	CBS 573.68	CBS	n.a.	-	n.a.
	commune	CBS 311.48	CBS	n.a.	-	n.a.
	corylophilum	CBS 321.48	CBS	n.a.	-	n.a.

	crustosum	CBS 499.73	CBS	n.a.	-	n.a.
	digitatum	DSM 62840	DSM	n.a.	-	n.a.
	expansum	DSM 62841	DSM	n.a.	-	n.a.
	glabrum	TMW 4.2027	TUM	n.a.	-	n.a.
	griseofulvum	TMW4.1543	TUM	n.a.	-	n.a.
	italicum	DSM 62846	DSM	n.a.	-	n.a.
	janthinellum	TMW 4.2318	TUM	n.a.	-	n.a.
	jensenii	TMW 4.2316	TUM	n.a.	-	n.a.
	nalgiovense	TMW 4.1371	TUM	n.a.	-	n.a.
	nordicum	BFE 487	TUM	n.a.	-	n.a.
	olsonii	TMW 4.1362	TUM	n.a.	-	n.a.
	purpurescens	CBS 223.28	CBS	n.a.	-	n.a.
	purpurogenum	CBS 286.36	CBS	n.a.	-	n.a.
	roqueforti	CBS 221.30	CBS	n.a.	-	n.a.
	roseopurpureum	TMW 4.1770	TUM	n.a.	-	n.a.
	rugulosum	TMW 4.1902	TUM	n.a.	-	n.a.
	stoloniferum	TMW 4.2280	TUM	n.a.	-	n.a.
	variabile	CBS 385.48	CBS	n.a.	-	n.a.
	verrucosum	CBS 603.74	CBS	n.a.	-	n.a.
	waksmanii	TMW 4.2317	TUM	n.a.	-	n.a.
Pseudogymnoascus	*destructans*	OT-38-2010	OT[14]	n.a.	-	n.a.
	pannorum	BBA 66108	BBA	n.a.	-	n.a.
Scopulariopsis	*acremonioides*	TMW 4.2366	TUM	n.a.	-	n.a.
Stachybotrys	*albipes**	ATCC 18873	ATCC[15]	n.a.	-	n.d.[16]
	bisbyi	ATCC 22173	ATCC	n.a.	-	n.a.
	chartarum	S 1352	LMU[17]	S	+[18]	n.a.
	chartarum	S 1344	LMU	n.r.[19]	+	n.a.
	chartarum	S 1349	LMU	S	+	n.a.
	chartarum	S 1418/1	LMU	S	+	n.a.
	chartarum	S BB2	LMU	S	+	+
	chartarum	S BO1a	LMU	S	+	+
	chartarum	S 1433	LMU	A	-	n.d.
	chartarum	S 1432	LMU	A	-	n.d.
	chartarum	S 1431	LMU	A	-	n.d.
	chartarum	S 1409	LMU	A	-	n.a.
	chartarum	S 1378	LMU	A	-	n.d.
	chartarum	S 1362	LMU	A	-	n.a.
	chartarum	S 1425	LMU	S	+	n.a.
	chartarum	S BT3	LMU	S	+	+
	chartarum	S BO2	LMU	S	+	+
	chartarum	S BO1b	LMU	S	+	+
	chartarum	CBS 324.65	TUM	H	-	n.d.
	chartarum	CBS 414.95	TUM	S	+	+
	chartarum	CBS 129.13	TUM	A	-	n.d.
	chartarum	S 1244	LMU	A	-	n.d.
	chartarum	R 24	LMU	S	+	+
	chartarum	S 1433	LMU	A	-	n.a.
	chartarum	S 1114	LMU	S	+	+
	chartarum	S6OW	LMU	H	-	n.a.
	chartarum	S 1432	LMU	A	-	n.d.

chartarum	S 1074	LMU	A	-	n.d.
chartarum	S 1286	LMU	A	-	n.d.
chartarum	S 41	LMU	H	-	n.a.
chartarum	S 42	LMU	H	-	n.a.
chartarum	S 43	LMU	H	-	n.a.
chartarum	S 1493/1	LMU	S	+	+
chartarum	S 1353	LMU	A	-	n.d.
chartarum	S 1341	LMU	H	-	n.d.
chartarum	S 1387	LMU	A	-	n.a.
chartarum	S 1342	LMU	H	-	n.d.
chartarum	S 1362	LMU	A	-	n.d.
chartarum	S 1335	LMU	H	-	n.d.
chartarum	S 1091	LMU	A	-	n.d.
chartarum	S 1166/2	LMU	S	+	+
chartarum	S 1285	LMU	H	-	n.d.
chartarum	S 1455	LMU	S	+	+
chartarum	S 1492	LMU	S	+	+
chartarum	S 1494	LMU	A	-	n.d.
chartarum	S 1589	LMU	A	-	n.d.
chartarum	S 3	LMU	H	-	n.d.
chartarum	S 4	LMU	S	+	+
chartarum	S 5	LMU	S	+	+
chartarum	S 9	LMU	S	+	+
chartarum	S 16St	LMU	S	+	+
chartarum	S 23St	LMU	S	+	+
chartarum	S 24It/B	LMU	A	-	n.d.
chartarum	S 35It	LMU	S	+	+
chartarum	S 48St	LMU	S	+	+
chartarum	H 47A	LMU	S	+	+
chartarum	H 47D	LMU	S	+	+
chartarum	IBT 7709	IBT	S	+	n.a.
chartarum	IBT 8709	IBT	A	-	n.a.
chartarum	IBT 8935	IBT	n.r.	+	n.a.
chartarum	Sp 2675	TUM	S	+	+
chartarum	TMW_4.685	TUM	A	-	n.a.
chartarum	TMW_4.674	TUM	A	-	n.a.
chartarum	TMW_4.689	TUM	S	+	n.a.
chartarum	TMW_4.675	TUM	A	-	n.a.
chartarum	TMW_4.678	TUM	A	-	n.a.
chartarum	TMW_4.680	TUM	A	-	n.a.
chartarum	TMW_4.682	TUM	A	-	n.a.
chartarum	TMW_4.684	TUM	A	-	n.a.
chartarum	S 1077	LMU	H	-	n.a.
chartarum	S 1333	LMU	H	-	n.a.
chartarum	S 1334	LMU	H	-	n.a.
chartarum	S 1339	LMU	H	-	n.d.
chartarum	R10	LMU	A	-	n.a.
chartarum	R09	LMU	A	-	n.a.
chartarum	R07	LMU	S	+	n.a.
chartarum	R06	LMU	S	+	n.a.

	chlorohalonata	S 48	LMU	n.a.	-	n.a.
	chlorohalonata	TMW_4.683	TUM	n.a.	-	n.a.
	chlorohalonata	TMW_4.676	TUM	n.a.	-	n.a.
	chlorohalonata	TMW_4.677	TUM	n.a.	-	n.a.
	chlorohalonata	TMW_4.688	TUM	n.a.	-	n.a.
	chlorohalonata	TMW_4.686	TUM	n.a.	-	n.a.
	chlorohalonata	TMW_4.672	TUM	n.a.	-	n.a.
	chlorohalonata	TMW_4.673	TUM	n.a.	-	n.a.
	chlorohalonata	IBT 40285	IBT	n.a.	-	n.d.
	chlorohalonata	H 47B	LMU	n.a.	-	n.a.
	chlorohalonata	H 47C	LMU	n.a.	-	n.a.
	chlorohalonata	H 42St	LMU	n.a.	-	n.a.
	chlorohalonata	S 44St	LMU	n.a.	-	n.a.
	chlorohalonata	S 24St/A	LMU	n.a.	-	n.a.
	chlorohalonata	S S01	LMU	n.a.	-	n.a.
	chlorohalonata	S 7	LMU	n.a.	-	n.a.
	chlorohalonata	S 1166/1	LMU	n.a.	-	n.a.
	chlorohalonata	CBS 413.95	TUM	n.a.	-	n.a.
	chlorohalonata	R04	LMU	n.a.	-	n.a.
	chlorohalonata	SBM29	LMU	n.a.	-	n.a.
	chlorohalonata	R01	LMU	n.a.	-	n.a.
	cylindrospora	ATCC 16276	ATCC	n.a.	-	n.d.
	dichroa	ATCC 18913	ATCC	n.a.	-	+
	elegans	DSM 66760	DSM	n.a.	-	n.a.
	kampalensis	ATCC 22705	ATCC	n.a.	-	n.d.
	microspore	ATCC 18852	ATCC	n.a.	-	n.a.
	oenanthes	ATCC 22844	ATCC	n.a.	-	n.d.
Trichoderma	*harzianum*	TMW 4.1502	TUM	n.a.	-	n.a.
	virens	CBS 344.47	CBS	n.a.	-	n.a.
Trichothecium	*roseum*	CBS 567.50	CBS	n.a.	-	n.a.
Zygosaccharomyces	*bailii*	DSM 70834	DSM	n.a.	-	n.a.
	bisporus	TMW 3.062	TUM	n.a.	-	n.a.
	rouxii	DSM 2531	DSM	n.a.	-	n.a.

*Anamorphic state of *Melanopsamma pomiformis*

[1] according to Ulrich et al. [34]

[2] according to the current study

[3] detection of macrocyclic trichothecenes according to [24, 34, 45, 46]

[4] Chair of Technical Microbiology, School of Life Sciences Weihenstephan, Technical University of Munich, Germany

[5] not applicable/not analysed

[6] negative result

[7] CBS strain collection at Westerdijk Fungal Biodiversity Institute, Utrecht, The Netherlands

[8] Deutsche Sammlung von Mikroorganismen und Zellkulturen, Darmstadt, DE

[9] Culture Collection of Fungi, Danish Technical University, Lyngby, Denmark

[10] Istituto Tossine e Micotossine da Parassiti Vegetali, CNR, Bari, IT

[11] Northern Regional Research Laboratory, Peoria (Illinois), USA

[12] Julius Kühn Institut, Bundesforschungsinstitut für Kulturpflanzen, Berlin, DE

[13] South African Medical Research Council, Tygerberg, SA

[14] Leibniz-Institut für Zoo- und Wildtierforschung, Berlin, Deutschland

[15] ATCC American Type Culture Collection, Manassas, USA

[16] not detected

[17]strain collection at Chair of Food Safety, Ludwig-Maximilian-University, Munich, Germany

[18]positive result

[19]no result
